# AI-assisted quantification of hypothalamic atrophy in amyotrophic lateral sclerosis by convolutional neural network-based automatic segmentation

**DOI:** 10.1038/s41598-023-48649-6

**Published:** 2023-12-06

**Authors:** Ina Vernikouskaya, Hans-Peter Müller, Francesco Roselli, Albert C. Ludolph, Jan Kassubek, Volker Rasche

**Affiliations:** 1 Department of Internal Medicine II, Ulm University Medical Center, Albert-Einstein-Allee 23, 89081 Ulm, Germany; 2 Department of Neurology, University of Ulm, Ulm, Germany; 3 German Center for Neurodegenerative Diseases (DZNE), Ulm, Germany; 4 Core Facility Small Animal MRI, University of Ulm, Ulm, Germany

**Keywords:** Amyotrophic lateral sclerosis, Brain, Biomedical engineering

## Abstract

The hypothalamus is a small structure of the brain with an essential role in metabolic homeostasis, sleep regulation, and body temperature control. Some neurodegenerative diseases such as amyotrophic lateral sclerosis (ALS) and dementia syndromes are reported to be related to hypothalamic volume alterations. Despite its crucial role in human body regulation, neuroimaging studies of this structure are rather scarce due to work-intensive operator-dependent manual delineations from MRI and lack of automated segmentation tools. In this study we present a fully automatic approach based on deep convolutional neural networks (CNN) for hypothalamic segmentation and volume quantification. We applied CNN of U-Net architecture with EfficientNetB0 backbone to allow for accurate automatic hypothalamic segmentation in seconds on a GPU. We further applied our approach for the quantification of the normalized hypothalamic volumes to a large neuroimaging dataset of 432 ALS patients and 112 healthy controls (without the ground truth labels). Using the automated volumetric analysis, we could reproduce hypothalamic atrophy findings associated with ALS by detecting significant volume differences between ALS patients and controls at the group level. In conclusion, a fast and unbiased AI-assisted hypothalamic quantification method is introduced in this study (whose acceptance rate based on the outlier removal strategy was estimated to be above 95%) and made publicly available for researchers interested in the conduction of hypothalamus studies at a large scale.

## Introduction

The hypothalamus has a crucial role in the regulation of the human body, involved in metabolic^1^, neuroendocrine^2^, immune^3^, and cardiovascular activity^4^. Detailed hypothalamic imaging has become of major interest to better characterize disease-associated clinical abnormalities including metabolism^5^. Several neurodegenerative diseases such as frontotemporal dementia (FTD)^6^ and Huntington disease (HD)^7^ are assumed to be related to hypothalamic volume atrophy. Specifically in amyotrophic lateral sclerosis (ALS), previous studies from different groups indicated that the total volume of the hypothalamus is substantially reduced in patients with ALS compared with controls^8–10^. ALS is traditionally conceptualized as a neurodegenerative disease affecting primarily the motor neurons whose degeneration is responsible for the severe motor phenotype of ALS. However, within the additional, non-motor symptoms of ALS with substantial impact on patient well-being and overall survival, proofs are available of a substantial hypermetabolic phenotype which predates, accompanies, and influences the clinical onset of ALS^11^. The cause of the hypermetabolic state in ALS has been subject to several mechanistic investigations, including the demonstration of an altered hypothalamic physiology^8–10^. The quantification of hypothalamic atrophy is thus an in vivo measure for the neuroimaging phenotype of neurodegenerative diseases like ALS and might be used for correlation analyses e.g., with the individual metabolic characteristics.

Due to the low size of the hypothalamus, the volumetric analysis in high resolution magnetic resonance imaging (MRI) is challenging. Due to limited image contrast in the vicinity of the hypothalamus, morphological landmarks require experience by the rater to be exactly determined in manual segmentation from MRI. As a consequence, results of different studies regarding hypothalamic volumetry show high variability during manual segmentation^12,13^. Moreover, manual segmentation is a very time-consuming and tedious procedure. Therefore, there is a need in neuroimaging for a reliable and unbiased technique to perform reproducible hypothalamic segmentation and volumetric analysis of large datasets, with a minimum of human intervention.

The success of deep learning methods in image classification has extended their use to solve more complex tasks including semantic segmentation^14^, which is the task of labeling pixels with a corresponding class of what is being represented. While there have been previous attempts at segmentation tasks, it was not until Ronneberger et al.^15^ with U-Net that a significant improvement in biomedical image segmentation performance was achieved^16^. The network is based on fully convolutional network (FCN) which consists of a contracting path (encoder) constituted by the general convolutional process to capture context and a symmetric expanding path (decoder) constituted by transposed convolutional layers that enables precise localization. Trained end-to-end from very few images, it outperforms the previously best methods and represents the state-of-the-art class of methods in terms of segmentation accuracy^15^. Because of its simplicity and effectiveness, U-Net has been widely adopted within the medical imaging community, improving the originally fully-convolutional network approach^17^. Since its inception in 2015, U-Net has seen many advancements in its architecture, e.g., U-Net architecture can be built with many different styles of backbone which is the architectural element which defines how the layers are arranged in the encoder network and determines how the decoder network should be built. Different classification networks as the backbone of the semantic segmentation network may show different performance^18^. There are several state-of-the-art pre-trained networks widely explored in the literature. Some famous examples in computer science applications are VGG16, ResNet50, Inceptionv3, and EfficientNetB0. VGG, ResNet, and Inception families are fundamental deep learning backbones already used for years for different tasks achieved excellent backbone-building performance^18^. EfficientNet networks are a recent family of architectures that have been shown to significantly outperform other networks in classification tasks while having fewer parameters^19^ and have been explored for medical image segmentation as an encoder^20^.

However, very few deep-learning based methods are available in the literature for hypothalamus segmentation on T1-weighted MR images. First, Rodrigues et al.^21^ implemented a fully automatic method based on max-tree to detect a bounding box around the hypothalamus in axial, sagittal, and coronal MR images and convolutional neural networks (CNNs) of 2D U-Net architecture to segment the hypothalamus within the detected region in each of three views with subsequent creation of a consensus from all three models’ outputs in order to help eliminate false positives. Their consensus model achieved a Dice coefficient of 0.77. Later, Billot et al.^22^ used a 3-D U-Net-based architecture with aggressive data augmentation to segment the hypothalamus and its subunits from one dataset with a Dice coefficient of 0.83 for the whole hypothalamus. Finally, Rodrigues et al.^23^ provided the first public benchmark composed of a diverse annotated dataset and achieved a Dice coefficient of 0.83 with the Teacher-Student-based model composed of modified EfficientNetB4 architectures for segmentation and correction.

The rationale of the current study is to present a fully automated approach to segment the hypothalamus on T1-weighted MRI scans and, based on this segmentation, to perform volumetric analyses of the hypothalamus in a large sample of healthy controls and in ALS patients to identify volume differences at the group level, i.e. atrophy associated with ALS. The method relies on application of CNN to segment both hypothalamus and intracranial volume (ICV) for hypothalamic volume normalization. The final validation of the AI-based segmentation was performed by comparison with volumetric results of an established manual delineation procedure, which has been used in previous studies and already obtained a high level of reproducibility in some hundred sporadic ALS cases and controls^9^.

## Results

### Performance of neural network hypothalamic segmentation

**Figure 1:**
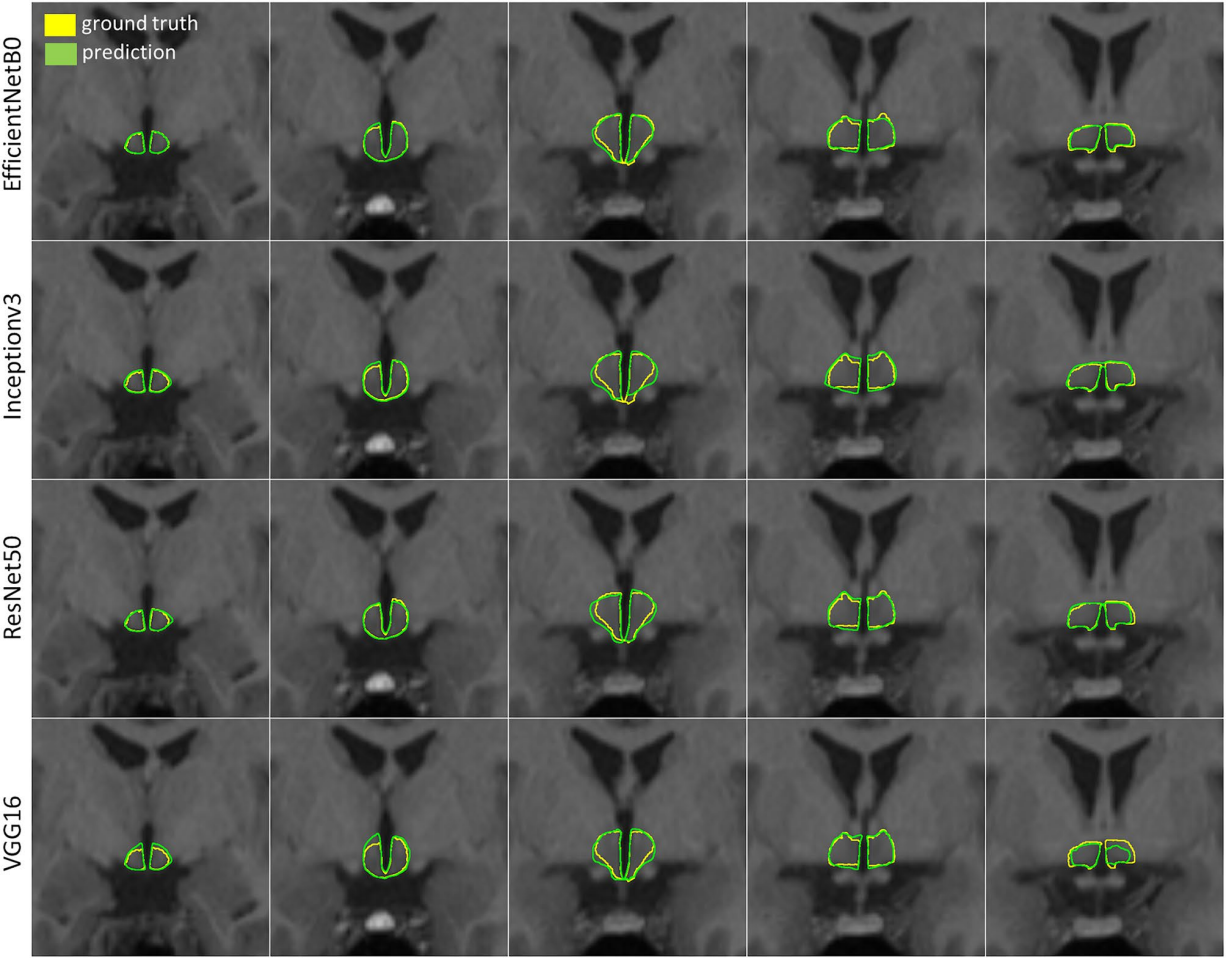
Comparison of segmentations (green contour) provided by four different U-Net models vs. ground truth (yellow contour) in five exemplary coronal slices of a single control test dataset.

Figure 1 shows the comparison of segmentations predicted by four different models with ground truth segmentations overlaid on the MR images in five exemplary coronal slices of a single control test dataset. Visual inspection of the automated segmentations shows that the overall anatomy of the hypothalamus is well learned by all networks. Disagreements between the ground truth and predicted segmentations were observed at the edge slices (anterior, posterior) where false pixels were predicted by all networks to some extent.

Table 1 summarizes the comparison of performance of hypothalamic segmentation by four investigated network architectures in the test dataset. All investigated network architectures achieved similar performance in terms of intersection over union (IoU) metric which measures the amount of overlapping between prediction and ground truth. The highest value was achieved for EfficientNetB0 (0.88). High Recall values for all models indicated that a high fraction of pixels that should be predicted as hypothalamus was also predicted as hypothalamus. The highest Precision, i.e., most of the pixels predicted as hypothalamus were true predictions, was achieved with EfficientNetB0 (0.87). Dice similarity coefficient was also highest for EfficientNetB0 (0.87) balancing Precision and Recall better than other models. The fastest prediction per image was achieved with EfficientNetB0, permitting segmentation of the whole hypothalamus (50 slices) in 1.43 s on a GPU.

**Table 1:** Comparison of performance of hypothalamic segmentation by four investigated network architectures in the test dataset.

	Ground truth	EfficientNetB0	Inceptionv3	ResNet50	VGG16
IoU	–	0.88 ± 0.03	0.87 ± 0.02	0.87 ± 0.02	0.85 ± 0.04
Precision	–	0.87 ± 0.05	0.79 ± 0.05	0.82 ± 0.05	0.82 ± 0.05
Recall	–	0.86 ± 0.05	0.93 ± 0.04	0.87 ± 0.04	0.85 ± 0.11
Dice	–	0.87 ± 0.03	0.85 ± 0.03	0.85 ± 0.03	0.83 ± 0.07
Prediction time per image/ms	–	29	31	35	42
Hypothalamic volume/cm^3^	0.82 ± 0.10	0.80 ± 0.10	0.96 ± 0.12	0.87 ± 0.11	0.85 ± 0.16
*p*-value	–	0.336^§^	1.189·10^–11§^	0.0003^§^	0.045^$^

^§^The reported p-values means that paired t-test was applied as statistics.

^$^Wilcoxon signed-rank test.

No significant difference between the ground truth volume and the volume segmented by EfficientNetB0 was observed, whereas all other networks significantly overestimated the segmented volumes.

**Figure 2:**
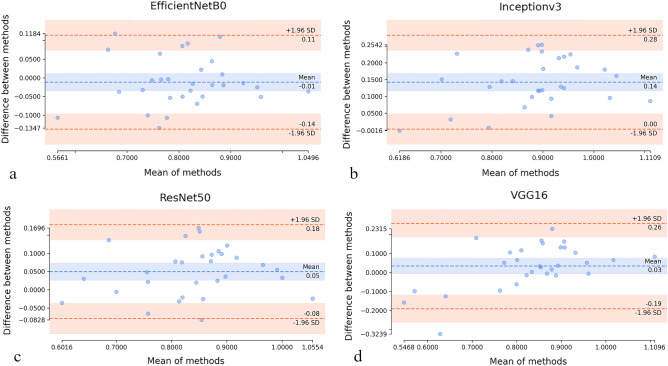
Bland–Altman plots assessing agreement between volumetric results provided by each of four investigated network architectures and ground truth: (**a**) EfficientNetB0, (**b**) Inceptionv3, (**c**) ResNet50, (**d**) VGG16. Mean difference and ± 1.96 SD limits of agreement are shown as blue and red dashed lines, respectively. Blue and red bands show confidence intervals on the mean difference and limits of agreement plotted over the 95% range, using the approximate method described by Bland and Altman.

Statistical results were confirmed by Bland–Altman plots (Fig. 2). The minimal mean difference between prediction and ground truth among the investigated networks was achieved with EfficientNetB0 (Fig. 2a) which only slightly underestimated the hypothalamus volumes (mean difference of − 0.01 cm^3^). The data points are equally distributed within a rather narrow range of the limits of agreement ([− 0.14, 0.11] cm^3^). Inceptionv3 consistently overestimated the hypothalamic volume (mean difference of 0.14 cm^3^ with 95% limits of agreement [0.0, 0.28] cm^3^) (Fig. 2b). ResNet50 also tends to overestimate the volumes (confidence interval of the mean difference above zero line) (Fig. 2c). Although the confidence interval on the mean difference for VGG16 contains the zero line (Fig. 2d), the range of limits of agreement is the largest ([− 0.19, 0.26] cm^3^), and an outlier falling outside of the confidence interval of − 1.96 SD limit of agreement is observed in these data, which may significantly impact the results by erroneously shifting the mean difference towards zero line.

Moreover, EfficientNetB0 has a much lower number of parameters, meaning faster training and lighter model.

### Volumetric analysis in the test group

After being re-trained on augmented data including images with limited contrast, EfficientNetB0 model achieved a Dice coefficient of 0.84 ± 0.03 in ALS and 0.86 ± 0.02 in controls. 95% HD was 0.82 ± 0.39 mm in ALS and 0.70 ± 0.38 mm in controls.

**Figure 3:**
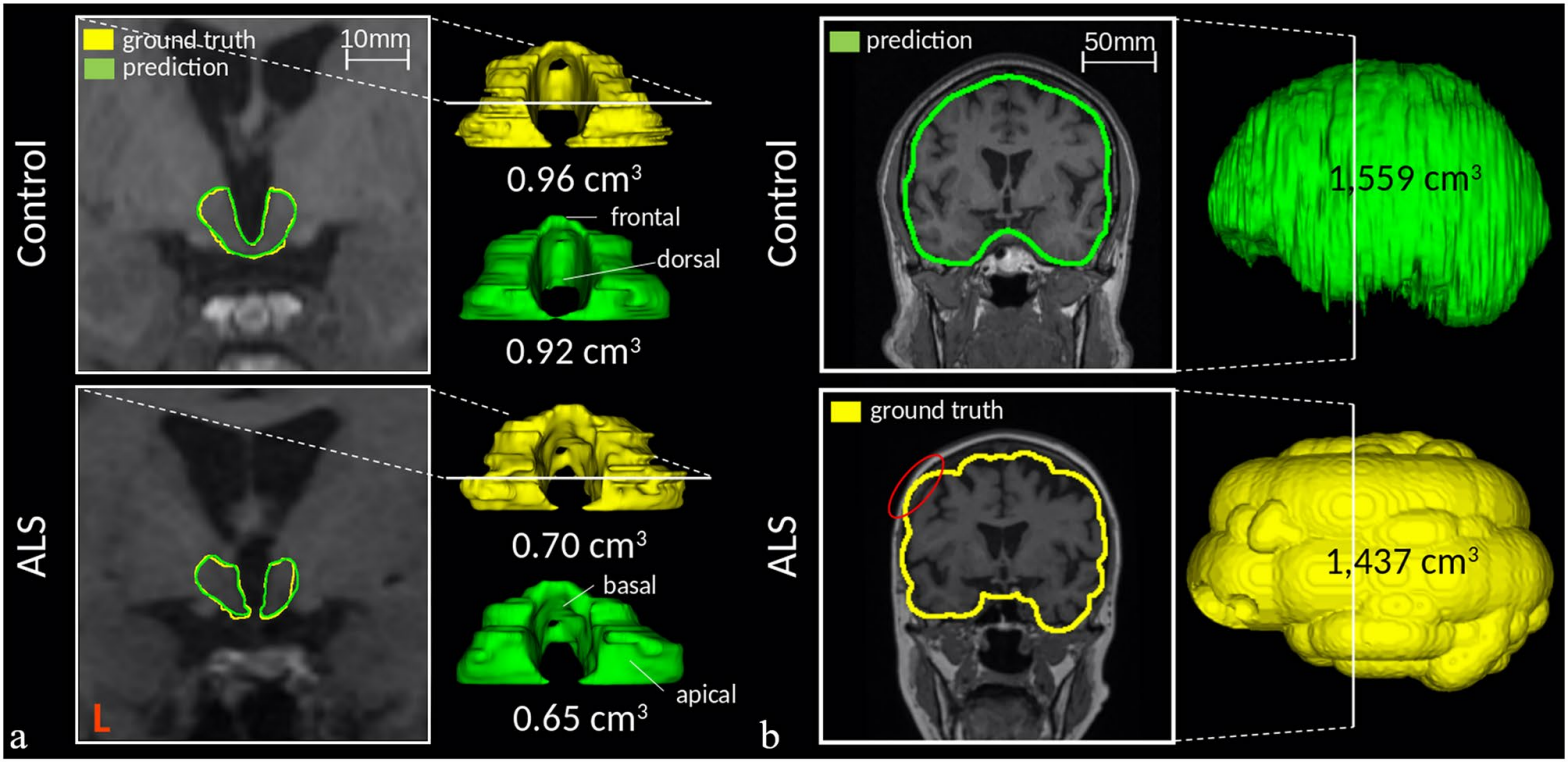
Predictions (green) and ground truth segmentations (yellow) of hypothalamus (**a**) and intracranial volume (**b**) as rendered 3-D models in a control (upper row) and an ALS patient (lower row) from the test dataset. MR image inlays with overlaid segmentation contours show localization of corresponding coronal slices. The smoothed surfaces of the ground truth intracranial volume are a result of the applied two-threshold technique for the manual segmentation. A partial overlap with other brain structures (dura) is marked in red.

Non-significant differences in calculated hypothalamic volumes between prediction and ground truth were observed both in ALS patients (0.80 ± 0.10 cm^3^ vs. 0.77 ± 0.09 cm^3^ in ground truth, *p* = 0.15) and controls (0.86 ± 0.07 cm^3^ vs. 0.86 ± 0.08 cm^3^ in ground truth, *p* = 0.76). Fig. 3a demonstrates qualitative results of hypothalamus segmentation in control and ALS patient as compared to ground truth.

To allow adjustments of the hypothalamic volumes between subjects with different head sizes, hypothalamic volumes in the test group were further normalized to intracranial volumes (ICV) (i.e., the sum of gray matter, white matter, and cerebrospinal fluid), which were automatically segmented using the same neural network approach. During generation of the training data for the ICV segmentation, the two-threshold technique did not work perfectly due to intensity inhomogeneities in the whole head image so that random errors at the borders (such as partial overlap with other brain structures) could appear (Fig. 3b as an example). Due to the intrinsic properties of the CNN approach, these non-associated random errors were not “learned” by the algorithm and the predicted volumes do not show these effects anymore.

**Figure 4:**
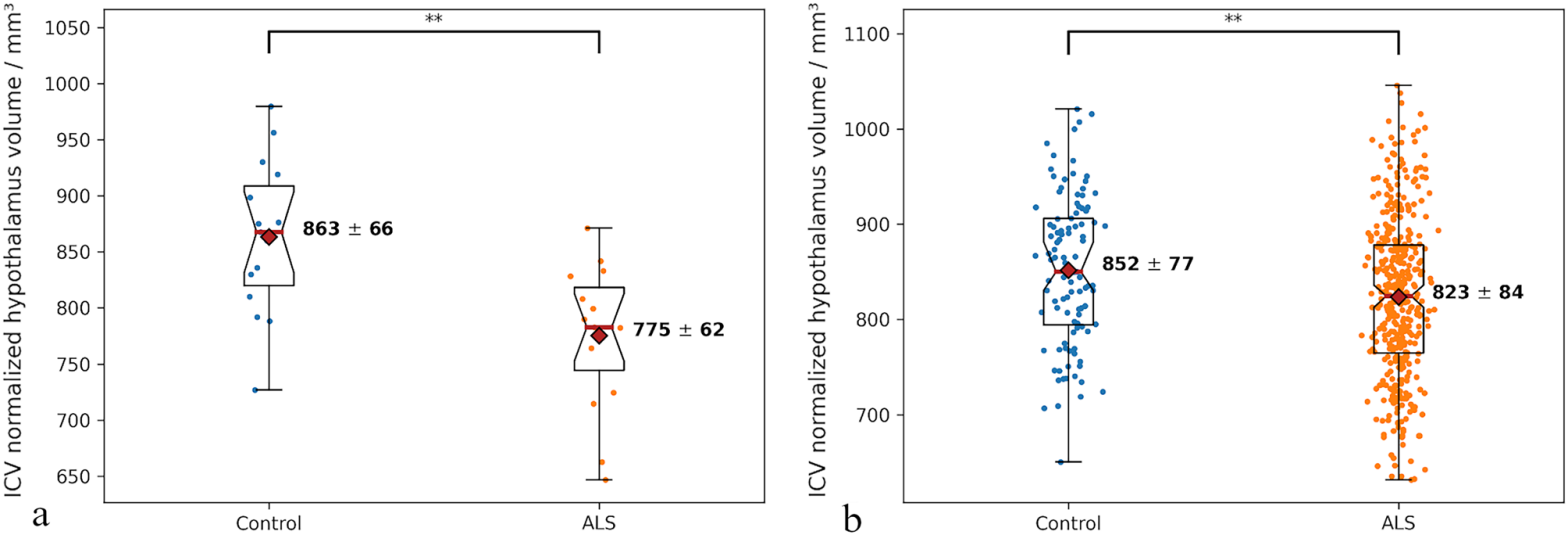
Comparison of automatically segmented hypothalamic volumes normalized to ICV in ALS vs. control groups in (**a**) test dataset containing ground truth data and (**b**) large neuroimaging dataset without the ground truth data. Median (red line), mean (red rhombus), and individual volumes (colored dots) are shown on the box-whiskers of ALS and controls. Double asterisk denotes statistical significance below 0.005 calculated applying unpaired t-test.

Normalized to the ICV (Fig. 4a), significant differences in hypothalamic volumes (− 10%, *p* = 0.0011) could be obtained between the ALS (775 ± 62 mm^3^) and control group (863 ± 66 mm^3^). Respective average volumes of manually segmented hypothalamus normalized to ICV comprised 870 ± 96 mm^3^ (controls) and 750 ± 66 mm^3^ (ALS) (ALS: − 14% vs. controls). Thus, differences between ground truth and automatically segmented hypothalamic volumes comprised 3% on average in the ALS group and did not exceed 1% in controls.

### Hypothalamic analysis in the group comparison

EfficinetNetB0 model trained without data augmentation could not perform any segmentation in 57 ALS cases and 2 control cases due to limited contrast of MRI scans, whereas the model trained with augmented data (with modified contrast distribution) in the training dataset could deliver segmentation results in 100% of cases.

According to box-whiskers plot for all data included in our dataset, three outliers above the upper limit of agreement in the ALS group were removed because the segmented ICV volume was highly underestimated (781 ± 191 cm^3^) in comparison to average ICV volume (1481 ± 165 cm^3^) in this group (*p* = 0.035). For 18 outliers below the lower limit of agreement in ALS group, the hypothalamus was significantly under-segmented (0.48 ± 0.16 cm^3^) as compared to average hypothalamus segmentation in this group (0.78 ± 0.11 cm^3^, p < 0.0005). In the control group, lower hypothalamus volumes (0.58 ± 0.09 cm^3^ vs. average 0.84 ± 0.09 cm^3^, *p* = 0.033) in combination with large ICV volumes (1714 ± 75 cm^3^) were detected as outliers after normalization in three cases; in two cases the ICV volume was underestimated (1258 ± 33 cm^3^ vs. average 1532 ± 139 cm^3^, *p* = 0.036), leading to outliers above the upper limit after normalization. With outliers, average hypothalamic volume in ALS group was calculated to be 812 ± 127 mm^3^ and in controls 847 ± 99 mm^3^ (ALS: − 4% vs. controls, *p* = 0.0009).

After outlier removal (Fig. 4b), a significant reduction in hypothalamic volume could still be achieved in the ALS group (823 ± 84 mm^3^) as compared to controls (852 ± 77 mm^3^) (ALS: − 3% vs. controls, *p* = 0.002).

In conclusion, based on the outlier removal strategy, the acceptance rate of the proposed automatic approach for hypothalamus segmentation with consecutive brain normalization was estimated to be above 95%.

## Discussion

Hypothalamic atrophy, together with alterations in hypothalamic peptides controlling energy metabolism, is known to be associated with metabolic derangements in ALS^24^. Given that this hypothalamic involvement can, like metabolic alterations in general, be regarded as a potential treatment target in ALS^8^, hypothalamus volume quantification might be developed as an imaging-based biological marker by unbiased, time-efficient approaches. However, segmenting the hypothalamus is very challenging due to its low size and low contrast in its vicinity where it is surrounded by grey matter structures. Although a growing number of neuroimaging studies in the literature aims to assess volume alterations of the hypothalamus^6,7,25–27^, only few have focused on implementing an automated method to reduce human variability and enhance studies’ robustness^23^.

This study has to be considered to include several strengths. In the first line, we have presented an artificial intelligence-based approach to automatically segment the hypothalamus (and the intracranial volume) in T1-weighted MR images of the brain by use of convolutional neural networks of U-Net architecture. U-Net has been developed primarily for image segmentation tasks and obtained a high utility within the medical imaging community. There are several variants which adopt an encoder-decoder architecture of U-Net, aiming to improve performance compared to the original fully-convolutional network approach. In this study, a comparison is performed using four significant U-Net variants on the same dataset to observe an effect on segmentation performance as well as trade-offs with respect to computational time and complexity.

Most data-driven methods are very susceptible to data variability: this challenge is especially apparent when applying deep learning to brain MRI, where intensities and contrasts vary due to acquisition protocol, scanner-, and center-specific factors^28^. Our data originated from the same center and had been acquired at the same scanner, but were heterogenous in terms of protocol, software release, and operator, since acquired over several years. Thus, our algorithm was complemented by data augmentation to implicitly regularize our trained network by making it robust against contrast variations and increasing generalization at inference. Our segmentation approach permitted extremely fast hypothalamus segmentation at inference (in less than 1.5 s on GPU) as compared to even semi-automated approaches requiring 20–40 min processing time per hypothalamus^29^. With the Dice coefficients of 0.86 ± 0.02 in controls and 0.84 ± 0.03 in ALS patients, the state-of-the-art automatic segmentation methods presented in the literature (0.77 from^21^ and 0.83 from^22,23^, respectively) could be improved. The predicted differences between ALS patients and controls were about 10% in the test group, whereas differences between manual and AI-based segmentation did not exceed 3% in this group.

Finally, we performed a volumetric analysis of the hypothalamus normalized to ICV in ALS patients vs. controls in a large neuroimaging dataset as a typical scenario for which our approach was intentionally developed for. Basically, our results were in line with previous independent MRI studies from different groups, reporting hypothalamic atrophy in ALS^9,10,30^, recently confirmed in a neuropathological study^31^. This significant hypothalamic volume reduction in ALS in comparison to controls at the group level could be confirmed in the current dataset. The predicted differences between ALS and controls in the large dataset comprised 3%. This value is highly significant, however, lower than previously reported^9,31^. This accuracy is sufficient for studies at the group level; however, in order to obtain robust clinical results at the individual level e.g., in clinical diagnostic procedures of a given disorder, further improvements should be necessary.

This study also has to be regarded in the context of its limitations. It might be regarded as a limitation that the technique did not work in all cases. Inaccuracy in ICV volume prediction of 5% leads to a maximum inaccuracy in hypothalamus volume calculation of about 10%. This value approaches the expected difference in the hypothalamic volume between ALS and healthy controls^9^. Therefore, outliers identified in predicted ICV volumes which fell out of the 5% range of average ICV volumes were considered to be outliers and were removed from the analysis, simultaneously defining the acceptance rate of AI-based approach. So, automatic segmentation of hypothalamus and/or ICV was rejected in 5 controls (out of 112) and 21 ALS (out of 432) cases, resulting in a rejection rate of the method below 5%. We identified as main reason for failure in hypothalamus segmentation the distortions of the MR images during pre-processing or due to breathing-related motion artifacts, especially in patients with high disease burden. This challenge can be tackled in the future by augmenting the training dataset with spatially deformed images. ICV segmentation failed in MR volumes with reduced contrast since such data were not part of the training. Generally, the challenge of contrast variation in MR images can, alternatively to the augmentation approach applied here, be addressed with z-score normalization of the data without the need for data augmentation. Better contrast can be obtained at a 3.0 T MRI scanner, potentially resulting in an improvement of the accuracy of segmentation. However, for the current study, some hundred T1-weighted MRI scans of MND patients as part of the standard clinical MRI protocol were available at 1.5 T. Future studies with the use of 3 T (or higher) should be performed. A final limitation of the current study is the use of data from a single imaging center which can lead to performance losses when predicting images from different datasets than those used in the training.

In conclusion, we present an AI-based technique for automated hypothalamus segmentation and volumetric analysis to be performed in an unbiased, reproducible manner and at a large scale. We applied this technique to study hypothalamic atrophy associated with ALS at the group level. Future work will focus on extending this automated analysis to applications to other neurological diseases, such as dementia syndromes (like FTD or Alzheimer’s disease), Huntington disease or Parkinson’s disease. To encourage other researchers to reproduce our results on their own datasets, we provide the source code as well as the trained models on GitHub: https://github.com/vernikouskaya/hypothalamus_segmentation.

## Material and methods

### Ethical approval

The ethics application includes the recording and the analysis of MRI data, irrespective of the analysis technique; no additional MRI scans have been performed for the current study. Previous studies on the analysis of MRI data have already been performed (^9,32^) and have been approved by the Ethics Committee of the University of Ulm (references #19/12 and #20/12) in accordance with the ethical standards laid down in the 1964 Declaration of Helsinki and its later amendments. Written informed consent was obtained from all individual participants included in the study.

### MRI dataset

Six-hundred-and-sixty-four T1-weighted whole head MRI datasets were acquired on a 1.5 T MRI scanner (Symphony, Siemens Medical, Erlangen, Germany) (Table 2A). Morphological data were obtained with a MPRAGE sequence (144 sagittal slices, no gap, 1.0 × 1.2 × 1.0 mm^3^ voxels, 256 × 192 × 256 matrix, TE = 4.2 ms, TR = 1600 ms), which is part of a standard clinical MRI examination protocol for patients with motor neuron diseases (MND). One-hundred-and-fifty-four healthy subjects without any neurological/psychiatric disease or other medical condition composed the control group. Five-hundred-and-ten patients with sporadic ALS were recruited in the outpatient and inpatient settings of the Department of Neurology, University of Ulm, Germany and composed the ALS group. One-hundred-and-twenty datasets (78 ALS patients and 42 controls) out of these groups were available with corresponding manual delineations of hypothalamus. After pre-processing based on a visual quality check, 12 ALS hypothalamic volumes were removed from the dataset due to limited contrast of the MR images. Thus, a subset of 108 hypothalamic volumes (50 slices each) were used for the training of different network architectures and the comparison of their performance. These data were randomly split on the subject level into training (47 ALS, 24 controls), validation (4 ALS, 3 controls), and previously unseen test (15 ALS, 15 controls) datasets, respectively, at a ratio of 66%/6%/28%, resulting in 3550 images for training, 350 images for validation, and 1500 images for test (Table 2B). Controls and ALS groups in the test sample were gender- and age-matched.

**Table 2:** Overview of available datasets and their splits during hypothalamic segmentation: (A) Total amount, (B) Network implementation, (C) Neuroimaging dataset.

			No. of subjects				No. of images	
			Control	ALS	All	Percentage all	Control	ALS
(A)	Total (w. and w/o GT)		154	510	664			
(B)	Network implementation	Training	24	47 (+ 12)	83	66% (69%)	3550 (27,350)	
		Validation	3	4	7	6%	350 (2250)	
		Test	15	15	30	28% (25%)	750	750
		Total (w. GT)	42	66 (+ 12)	108 (+ 12)	100%	5400 (31,100)	
(C)	Neuroimaging dataset	Test (w/o GT)	112	432	544		5600	21,600

Numbers in brackets show effect of adding 12 ALS datasets that have been discarded due to limited contrast during data augmentation and re-training of the network.

### Data-preprocessing and manual segmentation protocol of hypothalamus

**Figure 5:**
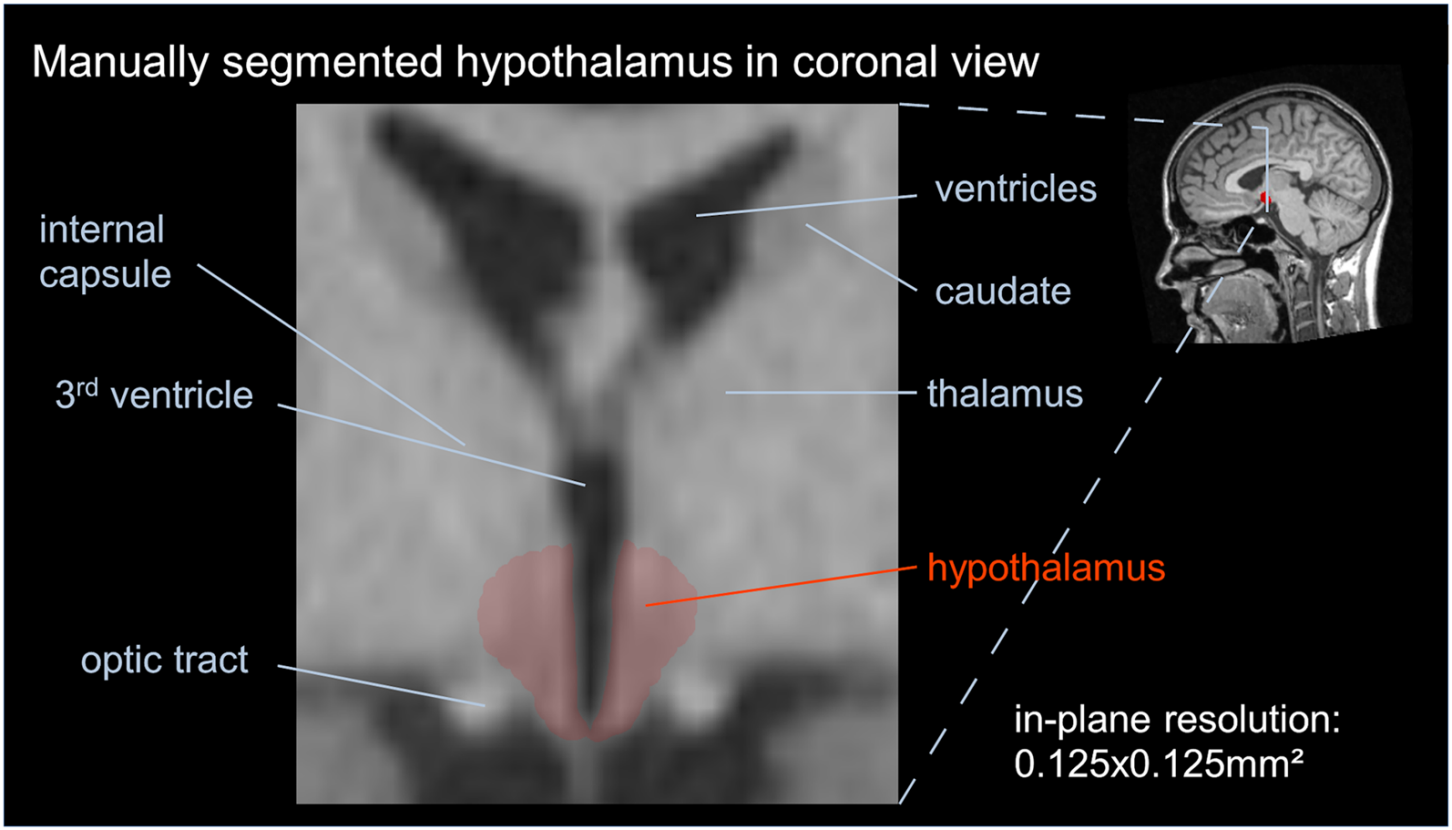
Exemplary coronal section with manually segmented hypothalamus in red and respective brain structures labeled. The inlay (central sagittal slice) shows the localization of this coronal section.

T1-weighted MRI data were used for manual delineation of the hypothalamus in the coronal plane in three-step pre-processing pipeline using the *Tensor Imaging and Fiber Tracking* (*TIFT*) software package expanded by a volumetric extension package^33^. The ground truth was obtained as a subsample from results of a previous analysis of some hundred sporadic ALS cases and 112 healthy controls^9^. First, the rigid body normalization of T1-weighted MRI data was performed along the anterior commissure (AC)—posterior commissure (PC) axis such that the coronal cutting plane was perpendicular with respect to the AC-PC axis to correct for individual tilt of the head and to minimize potential partial volume effects. Then, spatial upsampling was performed to improve the accuracy in visually identifying landmarks and hypothalamic borders. The hypothalamic section of each dataset was pre-selected in 50 slices of 0.5 mm thickness. Finally, manual delineation of the left- and right-hemispheric hypothalamus was performed using the highly reproducible technique adapted from Gabery et al.^12^, as previously described^9,34,35^. In short: boundaries in coronal sections were defined anterior when the optic chiasm was first seen to be attached to the ventral part of the septal area and posterior where the fornix appears to be merged with the mammillary nucleus. The hypothalamus was medially bounded by the third ventricle, the inferior border was defined by the junction of the optical chiasm for the anterior part, and by the border of the cerebrospinal fluid for the more posterior slices. The hypothalamus was laterally bounded by the diagonal band of Broca in the preoptic area, the internal capsule and the cerebral peduncle for the more posterior slices together with non-hypothalamic grey matter structures such as the fields of Forel on the most posterior slices. The optical tract was excluded from all slices. An appropriate visualization of the hypothalamic localization is provided in Fig. 5. With this manual delineation procedure, a high level of reproducibility was obtained with an intra-rater variability with a coefficient of variation < 4% and an intraclass correlation coefficient > 0.9 for inter-rater variability.

### Choice of network architecture

In the first stage of our work we compared the performance of four classification networks (VGG16, ResNet50, Inceptionv3, and EfficientNetB0) as backbones in the U-Net network for segmentation of the hypothalamus in the same experimental environment and with the same data.

All mentioned backbones weights were pre-trained on The ImageNet dataset^36^ to shorten the learning procedure, to speed up convergence, and to achieve high performance as compared to a non-pre-trained model. The 50 images representing the hypothalamic region had a size of 512 × 512 pixels with an in-plane resolution of 0.125 × 0.125 mm^2^. All models were trained on a GeForce GTX 1060 6 GB GPU for 25 epochs with early stopping with a batch size of 4 samples per pass. The loss function was the sum of the categorical Cross Entropy and Jaccard loss. Adaptive Moment Estimation (Adam) with the Keras default settings was used as the optimizer. The IoU was used as a metric to evaluate the model during training. Training was stopped when the validation loss was observed to have ceased improving for 10 consecutive epochs and the model with the lowest validation loss was chosen for prediction.

The segmentation performance of each CNN model was evaluated in terms of IoU. Further, true-positives ($TP$), i.e., the intersection between segmentation and ground truth; true-negatives ($TN$), i.e., part of the image beyond the union between segmentation and ground truth; false-positives ($FP$), i.e., segmented parts not overlapping the ground truth; false-negatives ($FN$), i.e., missed parts of the ground truth—were calculated for each volume to estimate the average Precision, Recall, and Dice coefficient. Average prediction time per image was assessed.

### Data augmentation

Because the contrast variation in the acquired MRI scans was a significant segmentation challenge, the training dataset was extended by the 12 ALS datasets excluded in the previous stage (resulting in 120 datasets) and the dataset was augmented by shifting the contrast of each image with some sampled values, such that the variability in the training set was similar to what is seen in real world clinical data, while preserving anatomical information, in order to make the network robust against contrast variations. That way, 27,350 images were obtained for training and 2250 images for validation (Table 2B). In the second stage of this work we re-trained the best model from the previous experiment on these data and repeated the evaluation in the same test dataset consisting of 15 ALS and 15 control datasets.

### Total intracranial volume—segmentation and normalization

We utilized the neural network with the best performed backbone used in previous experiments to automatically segment the ICV from original MRI volumes. To generate ground truth data of the ICV for training the network, manual delineation by visual intensity-based 3-dimensional marking of the ICV was performed by the *TIFT* software. In total, 10 ICV volumes (5 volumes from each test group from the previous experiment) were available for training (9 volumes, 4608 images) and validation (1 volume, 512 images) of the network.

Since no ICV ground truth data were available for the investigated hypothalamic test group, the evaluation was performed visually by 3-D reconstruction of the automatically segmented ICVs.

Finally, volumetric analysis was performed by calculating the normalized hypothalamus volume as:

1 $${{V}_{hypoth{al}_{norm}}=V}_{hypothal}/{V}_{ICV}*{V}_{{ICV}_{mean}\left(control\right)},$$

where $V$ denotes the automatically segmented volume of individual hypothalamus or ICV, respectively, and ${V}_{{ICV}_{mean}\left(control\right)}$ is the average ICV volume of controls.

### Application to hypothalamic volumetry in ALS

Then, we accessed the ability of the previously trained neural networks to reliably segment the hypothalamus (and consequently also the ICV) in a neuroimaging group level study (Table 2C), which represents a major application for the proposed method. Table 3 provides gender and age characteristics of ALS patients and controls in the neuroimaging dataset, as well as ALS-FRS-R score and disease duration for ALS patients.

**Table 3:** Overview of subject characteristics of the whole neuroimaging dataset. ALS-FRS-R—revised ALS functional rating scale^37^.

	Gender (m/f)	Age Mean ± std	Age Median (range)	ALS-FRS-R	Disease duration/months
ALS (N = 432)	257/175	62.6 ± 11.7	64 (21–93)	39 ± 7	17.6 ± 17.4
CON (N = 112)	56/56	61.2 ± 10.5	64 (24–82)	–	–
*p*-value	0.1	0.2	–	–	–

Since manual delineations were not available for the whole dataset, a quality check of the automated segmentation was performed based on the outliers detected in the predicted hypothalamus volume and ICV. The outliers were identified by applying interquartile range (IQR): any point outside the range [Q1 − 1.5*IQR; Q3 + 1.5*IQR] was considered to be an outlier.

### Statistical analysis

We used two evaluation criteria of the segmentation performance: first, Dice similarity coefficient measuring the overlap between predicted segmentation and ground truth and second, 95% Hausdorff distance which is similar to maximum HD, but based on the calculation of the 95th percentile of the distances between boundary points in the ground truth and prediction in order to eliminate the impact of a very small subset of the outliers.

The agreement in volume quantification between the ground truth and automatic segmentation provided by each model was analysed based on Bland–Altman plots. Comparison between the ground truth of the hypothalamic volumes and the hypothalamic volumes predicted by the networks was performed by applying a paired *t*-test or Wilcoxon signed-rank test as appropriate according to Shapiro–Wilk test for normality. The differences between ICV normalized hypothalamus volumes in the control and the ALS group were assessed applying unpaired t-test. A *p*-value < 0.05 was assumed statistically significant. The mean value and the standard deviation of the differences are reported.

## Data Availability

The original contributions presented in the study are included in the article, further inquiries can be directed to the corresponding author on reasonable request.
